# Long-Term Clinical and Radiological Outcomes of Cementless Total Knee Arthroplasty in Patients with Rheumatoid Arthritis

**DOI:** 10.3390/jcm15187091

**Published:** 2026-09-12

**Authors:** Filippo Calanna, Silvia De Martinis, Leonardo Clausetti, Alessia Invernizzi, Luca Tanel, Roberto Viganò, Alessandra Menon, Alessio Maione, Riccardo Compagnoni, Paolo Ferrua, Pietro S. Randelli

**Affiliations:** 1 U.O.C. I Clinica Ortopedica, ASST Gaetano Pini—CTO, 20122 Milan, Italy; filippo.calanna@asst-pini-cto.it (F.C.);; 2 Università Degli Studi di Milano, 20122 Milan, Italy; 3 U.O.C. II Clinica Ortopedica, ASST Gaetano Pini—CTO, 20126 Milan, Italy; 4 Department of Biomedical Sciences for Health, Università Degli Studi di Milano, 20133 Milan, Italy; 5 U.O.C Week Surgery, ASST Gaetano Pini—CTO, 20122 Milan, Italy

**Keywords:** knee, TKA, cementless, rheumatic, implants, arthroplasty

## Abstract

**Background:** Total knee arthroplasty (TKA) is an established treatment for end-stage rheumatoid arthritis (RA). Although cemented fixation has traditionally been preferred because of concerns regarding poor bone quality, advances in implant design and osseointegration have renewed interest in cementless fixation. This study evaluated the long-term clinical and radiological outcomes of cementless TKA in patients with RA. **Methods:** A retrospective single-center study included adult patients with RA who underwent primary cementless TKA between 2004 and 2021 using the same cruciate retaining implant. Clinical outcomes were assessed using range of motion (ROM), Visual Analog Scale (VAS), Oxford Knee Score (OKS), and patient satisfaction. Radiographic evaluation assessed implant fixation, while implant survivorship was analyzed using bilateral clustering via a Marginal Cox model analysis. **Results:** Seventy-five cementless TKAs performed in 52 patients were analyzed after a mean clinical follow-up of 10.8 ± 3.8 years. Mean ROM was 101° ± 26.5°, mean VAS score was 0.88 ± 1.84, and mean OKS was 40.3 ± 7.1. Overall, patients were satisfied or very satisfied in 89.6% of implants. Radiographic assessment showed no evidence of progressive radiolucent lines, osteolysis, implant subsidence, aseptic loosening, or malalignment. Only one revision was required because of periprosthetic joint infection, resulting in an implant survivorship of 98.3% at long-term follow-up. **Conclusions:** Cementless TKA demonstrated excellent long-term implant survival, durable radiographic fixation, and favorable clinical and functional outcomes in patients with rheumatoid arthritis. These findings support modern cementless fixation as a reliable and effective alternative to cemented TKA in carefully selected RA patients.

## 1. Introduction

Total knee arthroplasty (TKA) represents a well-established and effective surgical treatment for end-stage rheumatoid arthritis (RA), providing substantial pain relief, correction of deformity, and functional restoration [1,2]. Despite major advances in disease-modifying antirheumatic drugs and biologic therapies, a considerable proportion of patients with RA still progress to severe knee joint destruction requiring arthroplasty. Historically, RA has accounted for approximately 3–5% of primary TKAs in Western countries [3,4], although the burden of joint replacement in this population remains clinically relevant, particularly in younger patients with long disease duration.

However, RA presents unique biological and mechanical challenges for implant fixation. Chronic synovial inflammation, prolonged corticosteroid therapy, systemic osteopenia, and metaphyseal bone defects frequently compromise bone stock and may negatively influence implant stability [5].

For these reasons, cemented fixation has traditionally been recommended in RA patients, based on the assumption that immediate mechanical stability may compensate for impaired bone quality and reduce the risk of early loosening [6,7].

Nevertheless, cementless fixation has gained renewed interest over the past decades due to improvements in implant design, porous coatings, trabecular metal technology, and enhanced understanding of osseointegration biology. Modern cementless components aim to achieve durable biologic fixation while preserving bone stock and potentially facilitating future revisions [8,9,10]. Importantly, several mid- and long-term studies have challenged the historical preference for cemented fixation in RA [6,11,12,13,14,15].

Despite these encouraging data, the literature remains heterogeneous. Many available studies are retrospective, involve older implant designs, include mixed fixation techniques, or lack contemporary comparative data.

Therefore, the role of cementless fixation in TKA for RA remains a subject of debate. The purpose of this retrospective study was to evaluate the long-term clinical, radiographic, and survivorship outcomes of cementless TKA in patients with rheumatoid arthritis, in order to determine whether modern cementless fixation represents a reliable alternative to traditional cemented techniques in this specific population.

## 2. Methods

The study was designed based on the criteria of the Declaration of Helsinki and approved by the Institutional review board (IRB No. CTS-P-2026-09-LC).

Adult patients with rheumatoid arthritis who underwent primary cementless total knee arthroplasty (TKA) between 2004 and 2021 at a single center (ASST Gaetano Pini-CTO) were retrospectively reviewed. All procedures were performed by a single high-volume senior surgeon, and the same implant CR design (PROFIX^®^, Smith & Nephew, Memphis, TN, USA) was used in all cases. First introduced in 1994, PROFIX is an innovative knee arthroplasty design based on improved implant conformity, component fixation, patellar tracking, and surgical technique. The femoral (cobalt-chrome) and tibial (titanium) components are available with or without a porous coating augmented with a hydroxyapatite layer, allowing for cemented or cementless implantation, respectively; in the cementless configuration used in this series, fixation relies on biological osseointegration, whereby bone progressively grows into the porous surface to achieve durable fixation without polymethylmethacrylate (PMMA) cement, with initial mechanical stability provided by a press-fit insertion technique pending this secondary biological fixation. The following inclusion criteria were adopted: age > 18 years, diagnosis of rheumatoid arthritis, tricompartmental knee osteoarthritis KL grade III or IV, co-morbidities that do not contraindicate knee replacement, varus or valgus deformity < 20°, intact collateral ligaments, and availability of complete preoperative and postoperative clinical and radiographic documentation. Patients were excluded in case of varus or valgus deformity > 20°, previous joint infection, previous revision surgery, cemented components (or hybrid fixation), or incomplete documentation or data. Demographic and surgical data were collected from institutional digital and paper archives.

### 2.1. Clinical Evaluation

Clinical follow-up data were collected through telephone interviews. Patient satisfaction was assessed using a Likert scale, pain intensity was measured with the Visual Analog Scale (VAS), and functional outcomes were evaluated using the Oxford Knee Score (OKS). The OKS is a validated 12-item patient-reported outcome measure for total knee arthroplasty, with a total score ranging from 0 (worst outcome) to 48 (best outcome) [16].

Clinical evaluation, including knee range of motion (ROM), was assessed during a routine clinical follow-up visit at our hospital in all operated patients. During the subsequent telephone interview, patients confirmed that their range of motion had remained unchanged compared with the last clinical assessment performed at our institution.

### 2.2. Radiographic Evaluation

Postoperative radiographs were reviewed by musculoskeletal radiologists specializing in orthopaedic imaging. The presence or progression of radiolucent lines, osteolysis, component subsidence, implant loosening, and axial alignment were evaluated in order to identify a suboptimal fixation [17].

Radiological follow-up was defined as the interval between the date of surgery and the latest available radiographic assessment in the institutional archives.

### 2.3. Implant Survivorship

Postoperative complications requiring revision surgery, including periprosthetic joint infection and aseptic loosening, were recorded. Implant survivorship was defined as the absence of revision surgery during follow-up.

### 2.4. Statistical Analysis

Continuous variables are reported as mean ± standard deviation, median and interquartile range (IQR; first quartile [Q1]–third quartile [Q3]). Categorical variables are presented as counts and percentages. Prosthetic survivorship was evaluated using Kaplan–Meier survival analysis. To account for the lack of independence in patients undergoing bilateral total knee arthroplasty, continuous clinical outcomes (ROM, VAS, OKS) were analyzed using Generalized Estimating Equations (GEEs) with an exchangeable correlation structure. This model provided cluster-adjusted marginal means and robust standard errors. Implant survivorship was evaluated using a Marginal Cox Proportional Hazards model with a robust sandwich variance estimator, clustering the data by patient ID to calculate adjusted survival probabilities and properly widened 95% confidence intervals.

## 3. Results

### 3.1. Patient Selection and Demographic Characteristics

**Figure 1:**
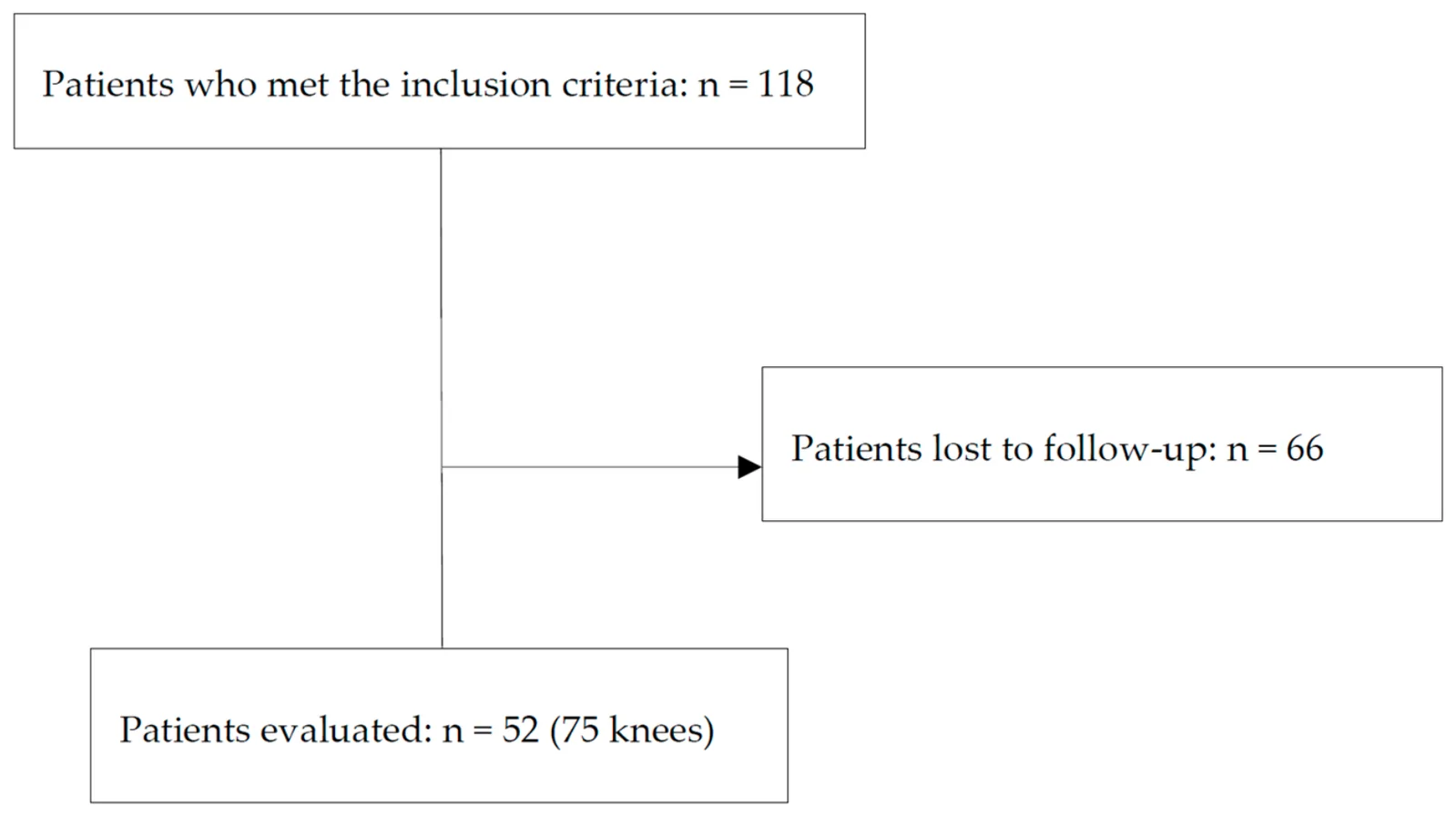
Flow diagram illustrating passage of patients through retrospective analysis.

A total of 118 patients met the inclusion criteria and were initially considered for the study. Of these, 66 patients could not be reached during follow-up, resulting in a dropout rate of 55.9%. In total, 52 patients were included in the final analysis (Figure 1).

Among these patients, 29 underwent unilateral TKA and 23 bilateral TKA, resulting in a total of 75 cementless TKAs included in the final analysis. Demographic data retrieved from institutional archives were verified during telephone interviews.

The study population showed a clear female predominance, with 58/75 implants (77.3%) performed in women. The distribution of the operated side was balanced, with 38 right knees (50.7%) and 37 left knees (49.3%). Mean age at surgery was 47.59 ± 13.52 years. Mean clinical follow-up was 10.83 ± 3.82 years (3–22), whereas mean radiographic follow-up was 2.79 ± 3.44 years (0–14). Mean age at final follow-up was 58.76 ± 12.85 years (Table 1).

### 3.2. Clinical and Functional Outcomes

Clinical and functional outcomes are summarized in Table 2. At final follow-up, patients achieved satisfactory medium- and long-term outcomes, with a mean ROM of 101.00° ± 26.45° (0–140). Pain levels were low, with a mean VAS score of 0.88 ± 1.84 (0–8). Functional outcomes were favorable, with a mean OKS of 40.25 ± 7.05 points (13–48). After applying the GEE model to account for bilateral clustering, the adjusted mean ROM was 102.10° (95% CI: 95.69–108.50). The adjusted mean VAS score was 0.95 (95% CI: 0.46–1.44), and the adjusted mean OKS was 40.49 (95% CI: 38.67–42.31).

During follow-up, only one prosthesis required revision surgery due to periprosthetic infection, occurring at approximately 6.3 years postoperatively. Adjusting for bilateral clustering via a Marginal Cox model, the overall implant survival rate was 98.6% at the final follow-up. The adjusted 95% confidence intervals reflect the effective sample size of the patient cohort rather than the total number of implants.

Overall patient satisfaction evaluated on the Likert scale was high, with patients being “satisfied” or “very satisfied” in 67/75 implants (89.6%). Only 2 cases (2.7%) were classified as dissatisfied.

### 3.3. Radiographic Outcomes

Radiographic evaluation demonstrated overall stable implant fixation after a mean radiographic follow-up of 2.79 ± 3.44 years. No significant progression of radiolucent lines, periprosthetic osteolysis, component subsidence, implant loosening, or axial malalignment was observed.

### 3.4. Implant Survivorship

During follow-up, only one prosthesis required revision surgery due to periprosthetic infection.

**Figure 2:**
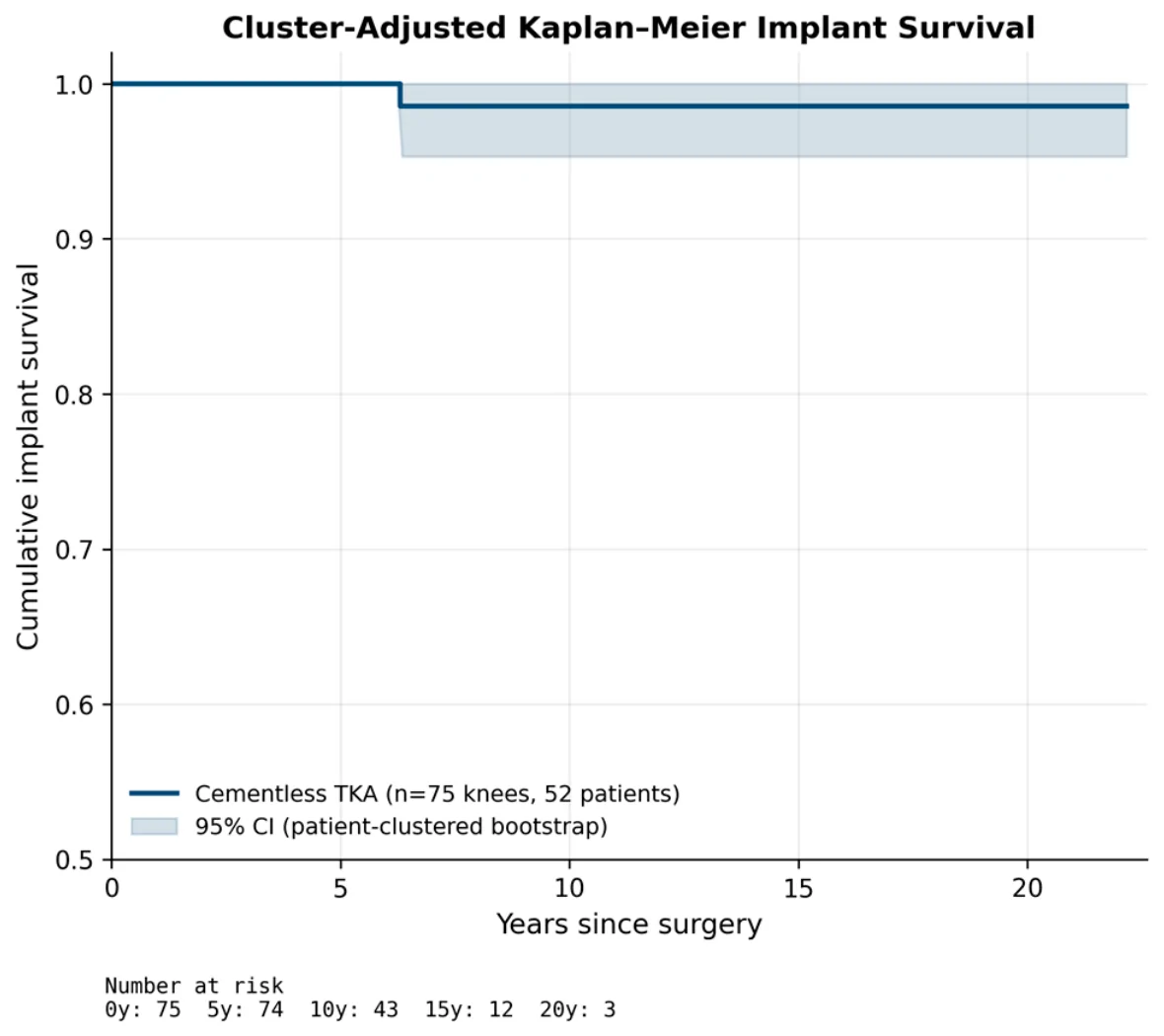
Cluster Kaplan–Meier curve of implant survival with 95% confidence intervals over 20 years after cementless TKA in RA patients. The solid line shows the recurrence-free survival using the Kaplan–Meier method, while the dotted lines indicate the 95% confidence intervals. The number of subjects at risk at each time point is reported below the graph.

Kaplan–Meier survival analysis demonstrated an implant survival rate of 98.3% at a mean follow-up of 10.8 years (Figure 2).

## 4. Discussion

The present study demonstrates that cementless total knee arthroplasty (TKA) in patients with rheumatoid arthritis (RA) yields satisfactory long-term survivorship and patient-reported outcomes, with an implant survivorship of 98.3% after a mean clinical follow-up of 10.83 ± 3.82 years. Importantly, no cases of aseptic loosening were observed during follow-up, and the only revision procedure was performed for periprosthetic joint infection. These results establish cementless fixation as a viable alternative to cemented techniques in selected RA patients.

The survival rate observed in this cohort was 98.3%. This compares favorably with published series on cementless TKA in RA. Woo et al. [14] reported a 96.8% survival rate at 15.5 years in 179 cementless TKAs in RA patients, while Yamanaka et al. [15] demonstrated 96.9% survival at 12 years using a cementless cruciate-retaining design. Hotfiel et al. [12] reported 97.1% and 95.6% survival at 10 and 15 years, respectively, in a hybrid cementless cohort with no cases of aseptic loosening. More recently, Buchheit et al. [6] documented a 97% survival rate at 6-year follow-up in cementless TKA for inflammatory arthritis. Taken together, the available evidence consistently supports a 96–98% medium-to-long-term survival for modern cementless implants in RA—a benchmark that the present series meets and slightly exceeds.

Importantly, the sole failure in this cohort was attributable to periprosthetic joint infection (PJI), with no case of aseptic loosening recorded over the entire follow-up period. This finding is clinically significant and reflects the broader epidemiological shift documented by Hauer et al. [18] in a large international registry analysis, which demonstrated a rising incidence of septic complications alongside a decline in aseptic loosening across modern TKA cohorts. The absence of aseptic loosening in our series is further supported by the radiographic data, which revealed no significant progression of radiolucent lines, periprosthetic osteolysis, or component subsidence—consistent with durable osseointegration achieved by modern porous-coated implant surfaces.

The theoretical concern that impaired bone stock secondary to chronic synovial inflammation, prolonged corticosteroid use, and systemic osteopenia might compromise osseointegration in RA patients [7,19] does not appear to be borne by the clinical evidence accumulated over the past two decades. Advances in implant surface technology such as the new designs of tibial keels, the introduction of 4 pegs on the implant–bone interface and the new materials developed by the industry—including highly porous titanium and trabecular metal coatings—have substantially enhanced primary stability and long-term biologic fixation, even in the context of reduced bone mineral density [10].

The present results contribute to a growing body of evidence challenging the historical paradigm of mandatory cemented fixation in RA.

The mean OKS recorded at final follow-up was 40.25 ± 7.05 points. This result is consistent with a satisfactory functional outcome: a threshold score of ≥40 has been validated as indicative of good prosthetic function with minimal impact on daily activities [20], and this benchmark was met by 72% of the implants examined. These results align with data reported by Qiao et al. [21] in a recent systematic review and meta-analysis comparing TKA outcomes in RA versus osteoarthritis (OA), which confirmed that RA patients consistently achieve lower absolute functional scores but demonstrate comparable or superior satisfaction rates relative to their OA counterparts.

The low mean VAS score (0.88 ± 1.84) observed at final follow-up reflects substantial pain relief compared with the severe preoperative pain typically reported in patients with end-stage rheumatoid arthritis, where mean VAS values of approximately 6.6 have been described [22]. Blevins et al. [23] documented a mean VAS decrease of −5.38 in RA patients following TKA—significantly greater than the −4.48 observed in OA patients—underscoring the particular efficacy of arthroplasty in addressing the nociceptive component of RA-related joint pain. In our series, 70.7% of patients were entirely asymptomatic at follow-up, a proportion in line with rates of 60–79% reported by Xu et al., Schrøder et al., and Yamanaka et al. [13,15,24], and attributable in part to well-controlled systemic disease activity in a modern DMARD-treated population.

The mean ROM was 101.00° ± 26.45°, which is sufficient for most activities of daily living, including stair climbing (≥90°) and rising from a chair (≥100°) [25]. However, it remains lower than the 110–120° typically reported after TKA in patients with osteoarthritis [26]. This difference is in our opinion due to the preoperative articular deformity characteristic of advanced RA, which constitutes a well-established predictor of postoperative ROM [27]. Notably, the two outlier cases with ROMs of 0° and 30° were both attributable to documented non-compliance with postoperative rehabilitation protocols, highlighting the critical role of structured physiotherapy in optimizing functional recovery after TKA [28,29].

The overall satisfaction rate observed in our cohort was 89.6% (“satisfied” or “very satisfied”). This is consistent with current benchmarks for TKA outcomes. Singh et al. [30] recently reported a satisfaction rate approaching 90% in a large contemporary TKA registry analysis. Notably, satisfaction rates following TKA in RA patients are reported to exceed those in OA patients, despite inferior absolute functional scores—a paradox well explained by the lower preoperative functional expectations and the disproportionate burden of pain that characterizes the RA population [23,31]. Kobayashi et al. [31] demonstrated in a matched cohort analysis that RA patients achieved superior satisfaction scores despite lower postoperative functional activity levels, a finding attributed to the alignment between preoperative patient expectations and postoperative pain relief.

The two cases classified as “dissatisfied” in our cohort were associated with persistent pain and unmet ROM expectations. These outcomes are consistent with data from Zhang et al. [32], whose meta-analysis confirmed that dissatisfaction after TKA in RA is predominantly driven by residual pain and limited functional recovery—factors closely linked to disease activity, psychological comorbidities, and rehabilitation adherence. The overall dissatisfaction rate of 2.7% in the present series falls well below the 10–20% range reported in the general TKA literature [30], further supporting the clinical value of arthroplasty in this population.

Radiographic evaluation at a mean follow-up of 2.79 ± 3.44 years revealed no evidence of significant radiolucent line progression, periprosthetic osteolysis, component subsidence, frank implant loosening, or axial malalignment. The relatively short mean radiographic follow-up represents a recognized limitation of the present study (discussed below). However, the absence of radiographic failure signals—combined with the 98.3% clinical survival rate and the lack of aseptic loosening events—collectively supports the durability of osseointegration achieved with modern cementless implant technology in RA patients. A notable strength of the present findings is that these excellent long-term outcomes and stable radiographic integration were achieved using a traditional cementless implant design characterized by a standard sintered porous coating. While modern advancements in highly porous titanium and trabecular metal technology were developed to further optimize early stability and bone ingrowth in compromised bone stock, our results demonstrate that standard cementless fixation is inherently viable in RA patients. The survivorship achieved with a traditional prosthesis strongly supports the underlying biological rationale for a cementless approach, suggesting that modern, advanced implants may yield equal or even superior longevity.

These findings are consistent with the biological rationale for cementless fixation in a younger, active RA population. While systemic osteopenia may theoretically impair primary stability, contemporary highly porous titanium and trabecular metal surfaces provide an osteoconductive scaffold that supports robust bone ingrowth even in the presence of reduced cortical density [8,9]. Furthermore, effective control of systemic inflammation through modern DMARD and biologic therapy may mitigate the adverse effects of active RA on periprosthetic bone remodeling—a factor not captured in older series that informed the original preference for cemented fixation [33,34].

RA presents a distinct clinical profile from OA that has direct implications for implant selection, surgical planning, and outcome interpretation. Chronic synovial inflammation, metaphyseal bone defects, soft tissue laxity, and the polyarticular and systemic nature of the disease contribute to greater surgical complexity and potential variability in postoperative outcomes compared to primary OA [5]. Moreover, sarcopenia—increasingly recognized as a relevant comorbidity in RA [35]—may impair quadricep recovery and delay functional rehabilitation, contributing to the lower absolute ROM and functional scores observed in this population relative to the OA controls.

Despite these challenges, the present results support the use of cementless TKA as a reliable treatment option in RA patients, particularly those of younger age and adequate baseline bone quality. The mean age at surgery of 47.59 ± 13.52 years in this cohort—substantially younger than the typical primary TKA population—reinforces the clinical relevance of bone stock preservation offered by cementless fixation, which may facilitate future revision procedures and reduce the complexity of potential re-implantation [8]. This consideration is especially pertinent given the expected longevity of implant function required in a younger patient population and the progressive nature of RA, which may necessitate revision surgery over an extended time horizon.

The growing availability and efficacy of disease-modifying agents—including conventional DMARDs, biologic therapies targeting TNF-α and IL-6, and JAK kinase inhibitors—have significantly altered the natural history of RA and reduced the proportion of patients progressing to end-stage joint destruction requiring arthroplasty [33,34]. However, for those patients who do require surgical intervention, arthroplasty remains the most effective treatment for pain relief and functional restoration [1,2,36]. The present data support cementless TKA as a component of the modern surgical armamentarium for this indication, complementing advances in pharmacological management.

## 5. Limitations

This study has several limitations that must be acknowledged. First, the retrospective, single-center design limits the ability to draw causal inferences and introduces inherent selection bias, particularly regarding the indication for cementless fixation. The absence of a concurrent cemented control arm precludes direct head-to-head comparison of fixation techniques, which would require a prospective randomized design. Second, the high dropout rate of 55.9% limits the representativeness of the analysed cohort and may introduce survivorship bias. This substantial loss to follow-up is primarily driven by the extended study timeline spanning from 2004 to 2021, combined with the inherently higher rates of medical comorbidities and competing mortality risks present in long-term RA cohorts. Furthermore, because our institution serves as a tertiary referral center, many patients travel from out of region for surgery and subsequently transfer their long-term clinical and radiographic care to local providers, significantly impeding remote institutional tracking. Third, the absence of systematic preoperative clinical score documentation (VAS, OKS, ROM) precludes a formal pre-to-post comparison of functional recovery, limiting outcome interpretation to the cross-sectional assessment at final follow-up. Fourth, the mean radiographic follow-up of 2.79 ± 3.44 years is shorter than the clinical follow-up and does not allow for reliable long-term assessment of periprosthetic bone remodeling or delayed radiographic loosening. Finally, clinical data were collected via telephone interview, which, while validated in large TKA registries, does not substitute for direct physical examination and a standardized in-person clinical assessment.

## 6. Conclusions

Cementless total knee arthroplasty in patients with rheumatoid arthritis demonstrated excellent long-term implant survivorship, satisfactory clinical and functional outcomes, and no cases of aseptic loosening at a mean follow-up of 11 years. These findings support cementless fixation as a reliable treatment option for patients with rheumatoid arthritis.

## Figures and Tables

**Table 1:** Demographic data of the study population.

	Total (N = 75)
Sex	
F	58 (77.3)
M	17 (22.7)
Age at surgery	47.59 ± 13.52 49.00 [37.00–57.00]
Age at follow-up	58.76 ± 12.85 59.00 [50.00–68.00]
Side operated on	
Right	38 (50.7)
Left	37 (49.3)
Years of follow-up	10.83 ± 3.82 11.00 [8.00–13.00]

Data from the sample of 75 prosthetic implants are reported as the number of prostheses (percentage) or as the mean ± SD and median [Q1–Q3]. SD: standard deviation; Q1: first quartile; Q3: third quartile.

**Table 2:** Assessment of clinical and functional outcomes.

	Total (N = 75)
**ROM**	101.00 ± 26.45 90.00 [90.00–120.00]
**VAS**	0.88 ± 1.84 0.00 [0.00–1.00]
**OKS**	40.25 ± 7.05 42.00 [38.00–44.00]
**Degree of satisfaction**	
Very dissatisfied	0 (0)
Dissatisfied	2 (2.7)
Indifferent	6 (8.0)
Satisfied	19 (25.3)
Very satisfied	48 (64.0)

Data are presented as the number of implants (percentage), or as the mean ± SD and median [Q1–Q3]. SD: standard deviation; OKS: Oxford Knee Score; Q1: first quartile; Q3: third quartile; ROM: range of motion; VAS: Visual Analogue Scale.

## Data Availability

All datas collected are available upon request and approval by the institution and the authors.
